# Strain-Modulated Flexible Bio-Organic/Graphene/PET Sensors Based on DNA-Curcumin Biopolymer

**DOI:** 10.3390/biom14060698

**Published:** 2024-06-14

**Authors:** Siva Pratap Reddy Mallem, Peddathimula Puneetha, Dong Yeon Lee, Sung Jin An

**Affiliations:** 1 Advanced Material Research Center, Kumoh National Institute of Technology, Gumi 39177, Republic of Korea; drmspreddy@kumoh.ac.kr; 2 Department of Robotics and Intelligent Machine Engineering, College of Mechanical and IT Engineering, Yeungnam University, Gyeongsan 38541, Republic of Korea; puneethaphd@gmail.com; 3 Department of Materials Science and Engineering, Kumoh National Institute of Technology, Gumi 39177, Republic of Korea

**Keywords:** DNA, biopolymer, graphene, strain sensor, gauge factor

## Abstract

In recent years, there has been growing interest in the development of metal-free, environmentally friendly, and cost-effective biopolymer-based piezoelectric strain sensors (bio-PSSs) for flexible applications. In this study, we have developed a bio-PSS based on pure deoxyribonucleic acid (DNA) and curcumin materials in a thin-film form and studied its strain-induced current-voltage characteristics based on piezoelectric phenomena. The bio-PSS exhibited flexibility under varying compressive and tensile loads. Notably, the sensor achieved a strain gauge factor of 407 at an applied compressive strain of −0.027%, which is 8.67 times greater than that of traditional metal strain gauges. Furthermore, the flexible bio-PSS demonstrated a rapid response under a compressive strain of −0.08%. Our findings suggest that the proposed flexible bio-PSS holds significant promise as a motion sensor, addressing the demand for environmentally safe, wearable, and flexible strain sensor applications.

## 1. Introduction

In recent years, flexible devices have attracted significant interest in future electronics/optoelectronics, which has notably boosted their commercial value [1]. These devices are continually being introduced in an array of advanced functional devices with sensing mechanisms, facilitating the emergence of wearable human–machine systems, soft robotics, and energy harvesting [2,3,4]. Strain-controlled flexible devices can be broadly classified into optical, resistance-based, and piezoelectric devices [5]. Among these, piezoelectric-based flexible devices stand out due to their rapid response time, superior sensitivity, and robust durability. Typically, an electrical strain sensor functions by converting mechanical energy into a specified quantity of electrical energy.

The development of strain-controlled sensors incorporating nanomaterials such as nanowires, nanotubes, nanoparticles, and thin films has attracted significant interest [6,7]. For instance, strain-controlled sensors comprising zinc oxide nanowires, graphene, and carbon nanotubes are potential alternatives for the fabrication of new strain-controlled sensors owing to their appealing properties [7,8,9,10]. In graphene-based strain-controlled sensors, the electrical conductance and principal vibrational frequency of graphene actively depend on its topological structure, which can be controlled by applying strain, making it useful for high-sensitivity strain detection [11,12]. Nanomaterials can serve as structural components and be modified to function as both multifunctional and multidirectional strain sensors at the nanoscale while exhibiting high gauge values. The electromechanical characteristics of these strain-controlled sensors exhibit outstanding functionalities compared to conventional strain sensors, attributed to the combined effects of their excellent electrical properties and large stretching moduli.

Recently, conduct polymers (CPs) have been used in the fabrication of sensors [13,14]. Specifically, they are used in different forms such as particles, films, fillers, and matrices. They are also used in the combination of CPs with additives and composites such as polyurethane, cotton, fabric, PDMS, Velostat, Ecoflex, and MXenes [13]. The development of a non-toxic and biocompatible multifunctional strain sensor that fulfills the requirements of high flexibility, mechanosensitivity, and robustness remains a challenge. Many biocompatible materials, including carbon dots, fluorescent proteins, and deoxyribonucleic acid (DNA), have been previously explored [15,16,17]. However, these biocompatible materials present limitations such as the extraction processing methods and protocols for carbon dots and fluorescent proteins. Nonetheless, the development of biocompatible strain-controlled sensors using DNA as the strain-sensing material remains a promising approach [18,19]. Moreover, it is possible to modify the electrical properties of thin-layered DNA-based biomaterials for applications in innovative electronic devices [20,21]. Because of their unique advantages, including molecular wires, variable nanoscale lengths, and self-assembly, DNA biomaterials are excellent alternatives for cutting-edge biocompatible device technologies [22]. Stemming from their wide energy bandgap (4.7 eV), DNA biomaterials exhibit diverse transport modes such as tunneling (super-exchange), long-range hopping (multi-step), and hopping (single-step) [23,24]. Compared to inorganic materials, DNA has many benefits such as light weight, low-cost preparation protocols, fabrication, and flexibility [25,26]. Several researchers have developed high-performance electronic devices based on DNA biomaterials [27,28,29]. DNA may act as a hole transport and electron-blocking agent at the interface of the heterojunction, realizing the conversion of detected photons into electron–hole pairs with a huge conversion efficiency [23]. Moreover, DNA-based strain-operated devices employing mechanical forces at the nano/micro scale in modern bioscience technology, such as attachable and movable sensors in the human body, have been recently investigated.

Curcumin, the most economically accessible material globally, is a naturally occurring yellow-orange compound extracted from the roots of Curcuma Longa. It finds widespread use in food spices, traditional medicines, and cosmetics in Asian countries [30]. Renowned for its potent anticancer, antitumor, antibacterial, and antioxidant properties [31], curcumin is also used as a chromophore that generates efficient luminescence in biohybrid light-emitting diode technology [29]. Researchers have extensively explored the incorporation of curcumin into hydrophilic or biocompatible polymers to produce bioactive polymer composites. The loading of curcumin onto polymeric materials to form electrospun nanofibrous scaffolds or mats has also been pursued [32]. Interestingly, the mechanical properties of electrospun materials mainly depend on the composition of the polymer matrix and the concentration of curcumin [33].

In this study, we fabricated a biopolymer-based strain sensor (bio-PSS) using a contemporary pure DNA biopolymer endowed with high feasibility, flexibility, and strain sensitivity. To fabricate the bio-PSS device, we utilized a pure DNA biopolymer extracted from salmon fish sperm and a solvent extract of turmeric (i.e., curcumin). To our knowledge, there are no existing reports on DNA-curcumin biopolymer sensors integrated into polyethylene terephthalate (PET) substrates for the fabrication of flexible bio-PSS devices.

## 2. Materials and Methods

### 2.1. DNA Extraction Protocols

**Figure 1:**
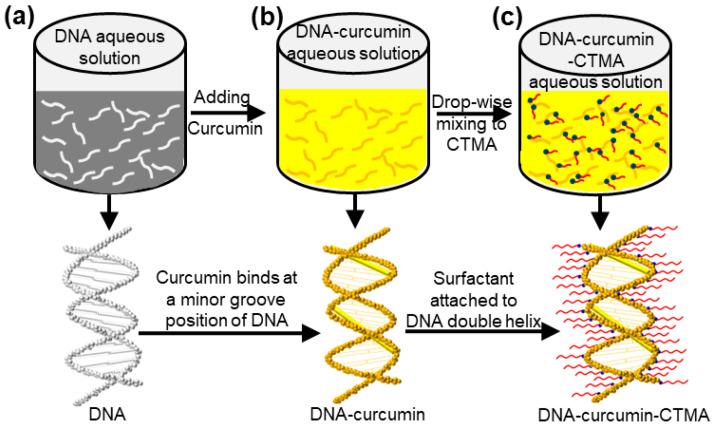
Processing protocols of DNA-curcumin-CTMA aqueous solution; (**a**) DNA; (**b**) DNA-curcumin; and (**c**) DNA-curcumin-CTMA.

Frozen DNA powder was extracted from salmon fish sperm, and cationic CTMA surfactant was purchased from Sigma-Aldrich. Curcumin powder, extracted from turmeric, was purchased from an Indian supermarket. To obtain the DNA-curcumin biopolymer, we followed the following protocol: 10 g of DNA powder was mixed with 1 L of deionized (DI) water in a glass beaker and stirred using a magnetic stirrer for 6 h to prepare an aqueous DNA solution (Figure 1a). In addition, curcumin (2 g) was mixed with 1 mL of dimethyl sulfoxide (DMSO, Sigma-Aldrich, Saint Louis, MO, USA). This mixture was added to an aqueous DNA solution and stirred for 6 h. During this process, a greenish-yellow aqueous solution of DNA-curcumin was obtained. Here, curcumin nanomolecules particularly bind at minor groove positions of DNA, as distinctly shown in Figure 1b. Similar characteristics such as curcumin binding to the DNA biomolecule with the minor groove were previously reported [27,34,35]. Using an ion-exchange reaction procedure, the CTMA surfactant was treated with an aqueous solution of DNA-curcumin and formed like DNA-curcumin-CTMA (Figure 1c). At this moment, the Na ions in CTMA combined with the DNA base pairs owing to the precipitation of DNA-curcumin-CTMA at the bottom of the glass beaker with the NaCl-containing solution. The combination of CTMA and DNA-curcumin precipitate was separated from the glass beaker and oven-dried overnight at 65 °C, yielding the bio-crystalline form of DNA-curcumin-CTMA precipitate. The product was then scraped and powdered. As the produced powder was insoluble in water and organic solvents, butanol was chosen as the solvent to prepare the target solution for the DNA-curcumin-CTMA biopolymer (hereafter DNA-curcumin biopolymer).

### 2.2. Graphene Transfer and Device Fabrication

**Figure 2:**
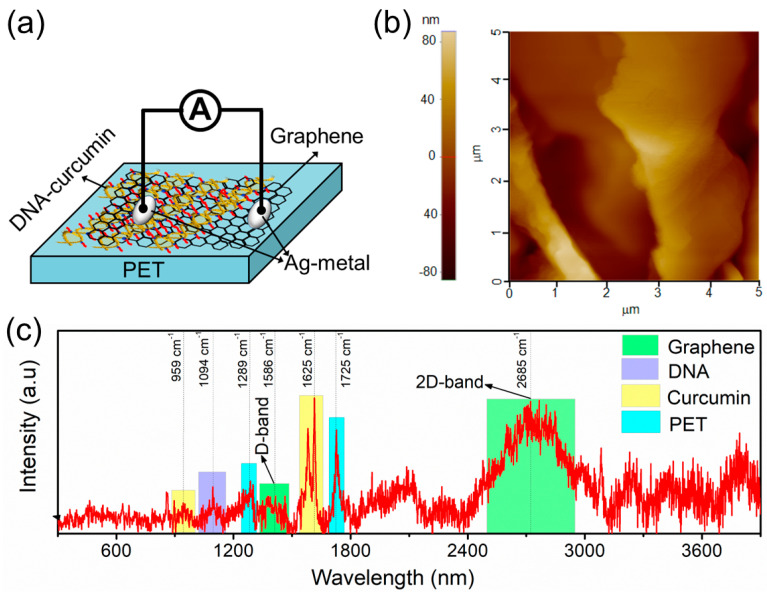
(**a**) Schematic illustration of the fabricated bio–PSS device; (**b**) two–dimensional SPM image; and (**c**) Raman spectrum of DNA–curcumin on the graphene/PET substrate.

A graphene layer was grown on a Si wafer (graphene/Ni/SiO_2_/Si) using the chemical vapor deposition (CVD) method. The graphene/Ni/SiO_2_/Si wafer was dipped in buffer-oxide-etch (BOE) solution to etch the SiO_2_ layer. The graphene/Ni layer was then rinsed in FeCl_3_ solution to etch the Ni layer. The PET substrate was then positioned in a glass container with DI water to transfer the graphene layer onto the PET substrate (i.e., the first graphene layer transfer). During this process, to further device fabrication, the DNA-curcumin biopolymer was spin-coated on the surface of the graphene layer (i.e., one portion of the graphene layer). The DNA-curcumin/graphene/PET wafer was then oven-dried for 3 h at 65 °C. Subsequently, Ag-metal contacts were applied to the surface of the graphene and DNA-curcumin layers. Thus, the desired Ag/DNA-curcumin/graphene/PET device was fabricated (Figure 2a).

### 2.3. Characterization Techniques

Scanning probe microscopy (SPM; NX-20 Park Systems, Suwon, Republic of Korea) was used to determine the thicknesses and morphologies of the samples. The spectra were obtained via Raman spectroscopy (inVia reflex, Renishaw, Wotton-under-Edge, UK) using a He/Ne laser at a wavelength of 532 nm. Transmittance spectra were recorded by UV-visible spectroscopy (PerkinElmer LAMDA, Waltham, MA, USA). The Fourier-transform infrared (FTIR, 80-Bruker, Vertex, Boston, MA, USA) spectrum was obtained in the spectral wavenumber range of 600–3000 cm^−1^. The structural phases were recorded using a sophisticated XRD (PANalytical, Westborough, MA, UK) instrument with a copper target material (Cu K_α_ radiation = 0.154 nm). The stress–strain curve (i.e., loading rate is ~0.16 mm/s) of the sample was obtained using a UTM (ORIENTAL, Hanshin Tech Co., Busan, Republic of Korea) instrument. The I-V and bending characteristics of the measured devices were systematically analyzed using a parameter analyzer (Keithley 4200-SCS, Tektronix, Beaverton, OR, USA).

## 3. Results and Discussion

SPM was used to determine the surface morphology of the DNA/graphene/PET, as illustrated in Figure 2b. The root-mean-square surface roughness of the sample was approximately 30.64 nm. The thickness of the multi-layer graphene layer (i.e., four layers) and DNA/graphene (i.e., three layers) were ~1.8 nm and ~150 nm on the PET substrate, respectively. XRD measurements of the DNA-curcumin/graphene/PET samples were performed to assess their structural phases. Raman spectroscopy was used to examine the spatial scattering modes of vibrations in a measured sample to detect the graphene, DNA, curcumin, and PET substrates. The Raman spectrum of the flexible DNA-curcumin/graphene/PET sample is presented in Figure 2c. The strong peaks at 1289 ± 1 cm^−1^ (C–O band) and 1725 ± 1 cm^−1^ (C=O) were correlated with the PET substrate. Similar characteristic PET peaks have been reported previously [36]. The two broad peaks corresponding to the G- and 2D-band were correlated with the graphene conducting layer. One peak at 1586 ± 1 cm^−1^ (G-band) appeared as a major in-plane vibrational mode. This mode occurs because of the two neighboring carbon atoms in a single layer of graphene. In addition, the second peak at 2685 ± 1 cm^−1^ (2D-band) is related to the doubly generated resonance linking of the two iTO phonons [37]. The two distinctive peaks identified at 959 ± 1 cm^−1^ and 1625 ± 1 cm^−1^ corresponded to the curcumin sample. These characteristics peaks were also observed by Nong et al. [38] and reported as νC=O vibration (959 ± 1 cm^−1^) and νC=O and νC=C vibration (1625 ± 1 cm^−1^) in curcumin molecules, respectively. The peak at 1094 ± 1 cm^−1^ corresponds to the symmetric stretching vibration mode of PO_2_^−^ in the DNA backbone and is considered an internal intensity standard for DNA content [39]. 

**Figure 3:**
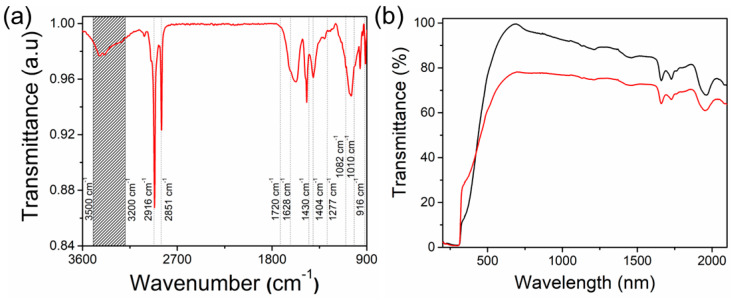
(**a**) FTIR and (**b**) UV–visible transmittance spectrum of DNA–curcumin/graphene/PET sample.

FTIR microscopy was performed to identify the stretching vibration interactions and harmonics in the DNA-curcumin/graphene/PET sample (refer to Figure 3a). The stretching vibrations of the bonds at 3200–3500 (O–H group), 1430 (C=C aromatic), and 1277 cm^−1^ (ν(C–O)) corresponded to curcumin [40,41]. The stretching bands at 2916 (CH_2_ asymmetric), 2851 (CH_2_ symmetric), and 916 cm^−1^ (phosphate-ribose skeletal motion) were ascribed to DNA components [42], and the bonds at 1725 (C=O) and 1406 cm^−1^ (O – H) originated from the graphene layer [42,43]. The bonds at 1082 (ester C=O stretching) and 1010 cm^−1^ (benzene-related in-plane vibration) were attributed to the PET substrate [7].

Figure 3b illustrates the transmission spectrum of the DNA-curcumin/graphene/PET sample. The strong transmission peak (at 683 nm) was found to have an optical energy bandgap value of 1.81 eV. We compared our sample with a sample without graphene, and the results showed an optical energy bandgap of 1.79 eV at a significant transmission peak of 693 nm. Therefore, the lower bandgap (~20 meV) of the DNA-curcumin/graphene/PET sample may be due to the graphene layer. A comparable reduction in the optical energy bandgap was observed for carbon-based materials [44,45]. 

**Figure 4:**
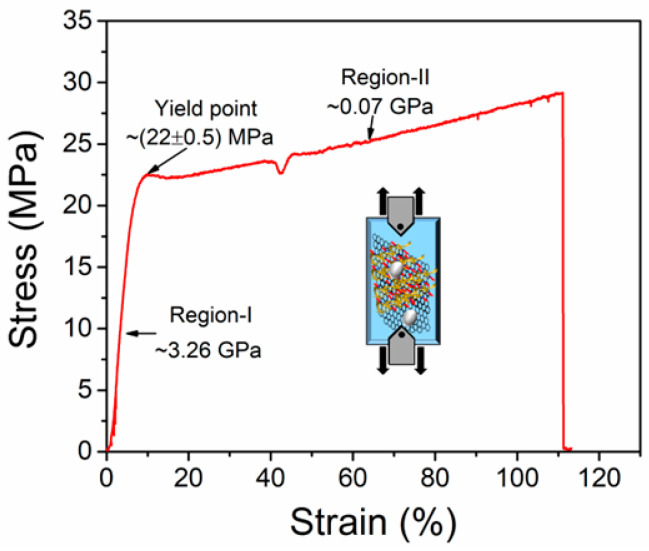
Stress-strain curve of DNA–curcumin/graphene/PET sample.

A UTM instrument was used to analyze the mechanical flexibility of the DNA-curcumin/graphene/PET sample. Here, both ends of the measured sample were held using the gripping inside the fixtures of the UTM machine as shown inset of Figure 4. Using a normal stretching strain-stress curve of the measured sample was carried out in a quasi-static state at a strain rate of 0.16 mm/s. However, the UTM measurements were performed under homogeneous uniaxial stretching conditions, and it was observed that the yield point occurred at approximately 22 ± 0.5 GPa, as shown in Figure 4. There were two distinctive plastic deformations in regions I (approximately 3.26 GPa) and II (approximately 0.07 GPa). Approximately 3.18 times the yield stress point was found at 111 ± 2% of the elongation break point. These results suggest that the mechanical and flexibility qualities were improved by applying strain. Consequently, the DNA-curcumin effect on flexible graphene/PET substrates is due to the enhancement induced by the strain. These features are important for flexible devices.

**Figure 5:**
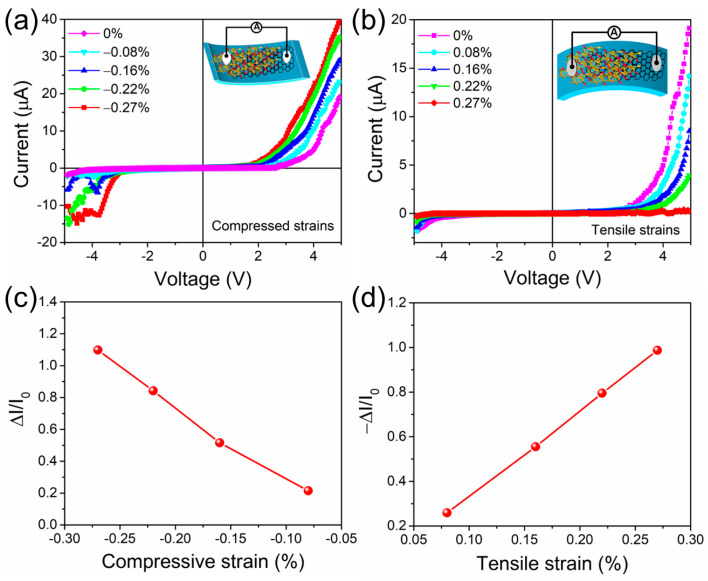
I–V data of flexible bio-PSS device with a sequence of (**a**) compressed and (**b**) tensile strains (inset shows the corresponding strain configurations). Relative deformations in current when the bio–PSS device was loaded along the (**c**) compressed and (**d**) tensile directions.

Figure 5a,b presents the I-V curves under initial and distinct strains (i.e., compressive and tensile directions) for the biopolymer-based flexible DNA-curcumin/graphene/PET strain sensor (bio-PSS). In Figure 5a,b, the output current of the sensor gradually increases with an increasing applied compressive strain and decreases with an increasing applied tensile strain. To evaluate the real-time working mechanisms and control of the flexible bio-PSS with applied strain, we assessed the injection current and strain of the device in the steady state (I_0_), change-in state (ΔI = I_C_ − I_0_), and relative deformation state (ΔI/I_0_ = I_C_ − I_0_/I_0_), where I_C_ and I_0_ are the initial and applied strains in the compressive and tensile directions, respectively. The correlation between ΔI/I_0_ and the applied strains in the compressive and tensile directions is shown in Figure 5c,d. These relationships were predicted based on the I-V characteristics (Figure 5a,b).

**Figure 6:**
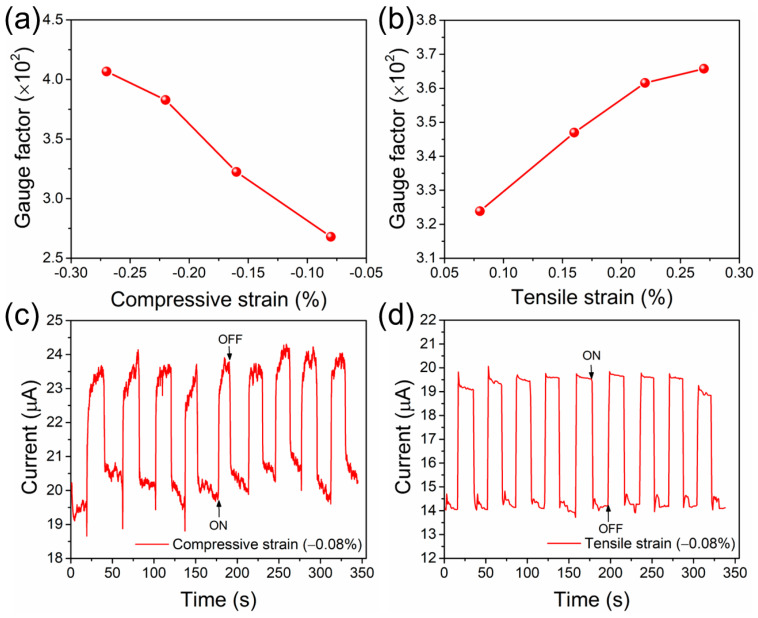
Gauge factor of flexible bio-PSS device evaluated at the applied bias of 5 V under different (**a**) compressive and (**b**) tensile directions. Time–dependent performance of the flexible bio-PSS under (**c**) compressive and (**d**) tensile loads.

The gauge factor is often used to describe the sensitivity of a strain sensor and can be evaluated as (ΔI/I_0_)/ε, which is the relative deformation in the current divided by the applied strain. Figure 6a,b shows how the applied strains in the compressive and tensile direction loads caused an increase in the gauge factor values. The evaluated gauge factors for the compressive strains of −0.08%, −0.16%, −0.22%, and −0.27% were 268, 322, 383, and 407, respectively, and 323, 347, 362, and 366 for the predicted tensile strains of 0.08%, 0.16%, 0.22%, and 0.27%, respectively. The measured strain gauge values were higher than the standard gauge values (i.e., 2) [46,47]. This illustrates that the strain gauge values of 268 (2^8.06^), 322(2^8.33^), 383(2^8.58^), and 407 (2^8.67^), which were 8.06, 8.33, 8.58, and 8.67 times higher than the traditional gauge value, were determined by the induced compressive strains of −0.08%, −0.16%, −0.22%, and −0.27%, respectively. Furthermore, the gauge factor values of 323 (2^8.33^), 347(2^8.44^), 362(2^8.5^), and 366 (2^8.52^) were obtained for the tensile strains of 0.08%, 0.16%, 0.22%, and 0.27%, respectively, which were 8.33, 8.44, 8.5, and 8.52 times higher than the conventional value. 

In actuality, the gauge factor values are dependent on an applied bias and the strain to control the piezoelectric effect. At a certain value of combined applied bias and strain, the pathways with low activation energy are already conductive (i.e., high current) and cannot be changed with an increasing strain. The values of the gauge factor are restricted due to certain strains already having reached a high conductive state. Further, the gauge factor value influences the materials’ characteristics such as the gauge factor of the graphene layer that ranges from 10 to 15, which is dependent on the number of graphene layers (1–5 layers) [48]. Furthermore, a DNA/graphene/GaN/PEN hybrid device demonstrates a high gauge factor of 898 [45]. Moreover, we did not use semiconductor materials in our present device (DNA/graphene/PET) and attained a high gauge factor of 407. This is the first approach we have used in which no semiconductor material is used. When compared to the current results, we will obtain higher gauge factors in future devices. 

Figure 6c,d illustrates the features of the response findings of the flexible bio-PSS under compressive and tensile strains. The current versus time plot with applied strains under compressive and tensile loads has a close resemblance in shape, indicating that the bio-PSS responded quickly and well. At an applied compressive strain of −0.08%, the approximate response time was 0.8 s. Further, at an applied tensile strain of 0.08%, the response time of the sample was 1 s. Thus, based on these strain outcomes, the bio-PSS is a promising active sensor that could meet the needs of the development of wearable/flexible sensors in the future.

## 4. Conclusions

In this study, we introduced a biocompatible material, DNA-curcumin, as a flexible strain-sensitive layer on a graphene/PET substrate using innovative and bio-inspired cutting-edge technology. The utilization of DNA-curcumin provides a cost-effective, tunable, and feasible approach to fabricating a biopolymer-based piezoelectric strain sensor (bio-PSS). Our proposed bio-PSS demonstrates highly sensitive, super mechanical behaviors while enabling bending functionalities. Specifically, it displayed compelling sensitivity, boasting a strain gauge factor 8.67 times greater than that of a conventional metal gauge under an applied compressive strain of −0.08%. Moreover, the bio-PSS demonstrated a rapid response time of 0.8 s under the same compressive strain. According to our analysis, the strain-modulated flexible bio-PSS based on biopolymer materials, such as DNA-curcumin, presents significant commercial viability for future applications in wearable/flexible electronics such as biomedical devices, soft robotics, and artificial intelligence.

## Data Availability

The data are available upon reasonable request from the corresponding author.
